# Minimally invasive aortic valve replacement using a bioprosthesis in a young adult with Wunderlich syndrome and bicuspid aortic valve

**DOI:** 10.1093/jscr/rjaf822

**Published:** 2025-10-20

**Authors:** Santiago Orozco Martinez, Manuela Orozco Martinez

**Affiliations:** S.E.S Hospital Universitario de Caldas, Calle 46 #25-71, Manizales, Caldas, Colombia; Universidad de Manizales, Caldas, Colombia

**Keywords:** aortic valve insufficiency, minimally invasive surgical procedures, heart valve prosthesis, young adult, bioprosthesis

## Abstract

We report the case of a 20-year-old male with a history of Wunderlich syndrome and prior partial nephrectomy, who was referred for cardiovascular surgery evaluation due to severe aortic regurgitation. Transthoracic echocardiography revealed bicuspid aortic valve type 1 with right–left cusp fusion and cusp prolapse, preserved left ventricular function (LVEF 55%), and marked left chamber enlargement. Given the patient’s history of major bleeding, a mechanical prosthesis was contraindicated. He underwent minimally invasive aortic valve replacement through upper mini-sternotomy with implantation of an Inspiris Resilia® #25 bioprosthesis and aortic reconstruction with pericardial patch. The postoperative course was uneventful, with adequate hemodynamic stability, no major complications, and early discharge. This case highlights the importance of individualized prosthesis selection in young patients with contraindication to anticoagulation, and the role of minimally invasive approaches in reducing morbidity and hospital stay while enabling future Valve-in-Valve strategies.

## Introduction

Aortic regurgitation (AR) is one of the most prevalent valvular heart diseases. Its etiologies can be classified into primary causes involving the valve cusps and secondary causes associated with abnormalities of the supporting structures. The most frequent cause is atherosclerotic degeneration, followed by congenital defects, particularly bicuspid aortic valve (BAV), which affects ~0.5%–2% of the general population [1]. BAV predisposes individuals to both aortic stenosis and regurgitation, as well as dilation of the aortic root and ascending aorta, increasing the severity of AR [2].

Approximately 35% of patients with BAV will develop complications, including valve dysfunction, endocarditis, aortic aneurysm, or dissection—the most common being valve dysfunction due to stenosis or regurgitation [3, 4]. Recent evidence has shown that the lifetime risk of complications in BAV patients exceeds 35%, and nearly 70% of individuals will ultimately require an aortic valve intervention during their lifetime [5]. The mechanisms of AR include cusp prolapse, infective endocarditis, myxomatous degeneration, or functional causes secondary to root dilation [3].

In young patients requiring aortic valve replacement, prosthesis selection poses a clinical challenge. Mechanical valves provide long-term durability but carry a significant burden due to lifelong anticoagulation, with associated thromboembolic and bleeding risks, difficulty maintaining therapeutic INR levels, and contraindications in pregnancy. Bioprosthetic valves avoid anticoagulation but are more prone to degeneration, necessitating future reintervention [6, 7].

Recent evidence supports the use of minimally invasive techniques, such as upper mini-sternotomy, which provide effective access while reducing perioperative morbidity. Adequate preoperative planning is essential to determine anatomical suitability, considering the position of the aorta relative to the sternum, pulmonary valve, and angulation for proper visualization and access [8, 9].

## Case presentation

A 20-year-old male with a medical history significant for dengue fever and spontaneous retroperitoneal hemorrhage secondary to renal rupture (Wunderlich syndrome), treated with partial left nephrectomy and endovascular embolization, was referred for cardiovascular surgery evaluation. Transthoracic echocardiography demonstrated severe AR due to BAV type 1 with right–left cusp fusion and cusp prolapse, preserved left ventricular ejection fraction (55%), end-diastolic volume of 67 ml, and left chamber enlargement consistent with valvular heart disease. An anterior mitral leaflet aneurysm was also observed without dysfunction.

On admission, he was hemodynamically stable: blood pressure (BP) 140/62 mmHg, HR 80 bpm, with no signs of heart failure or respiratory distress. Physical examination revealed a grade II/IV systolic murmur at the aortic area. Chest CT showed remnants of the thymic gland, a calcified granuloma in the right middle lobe, and cardiomegaly due to left chamber enlargement. Blood tests, electrolytes, infection panel, renal function, and thyroid-stimulating hormone (TSH) were all within normal limits (Table 1).

**Table 1:** Laboratory test results.

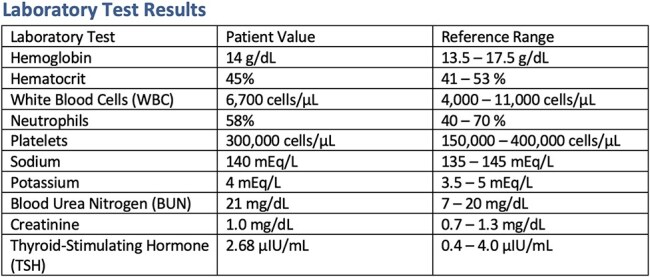

Cardiovascular surgery assessment confirmed the indication for early aortic valve replacement, in accordance with current guideline criteria for severe AR. The patient presented with massive regurgitation, left ventricular enlargement (end-diastolic volume 67 ml) despite preserved ejection fraction, and was at high risk of progression to heart failure and adverse outcomes [10]. Given his history of major bleeding, a mechanical valve was contraindicated due to the need for anticoagulation. A bioprosthetic Inspiris Resilia® 25-mm valve was selected, recognized for its enhanced durability and compatibility with future valve-in-valve interventions [11, 12].

**Figure 1:**
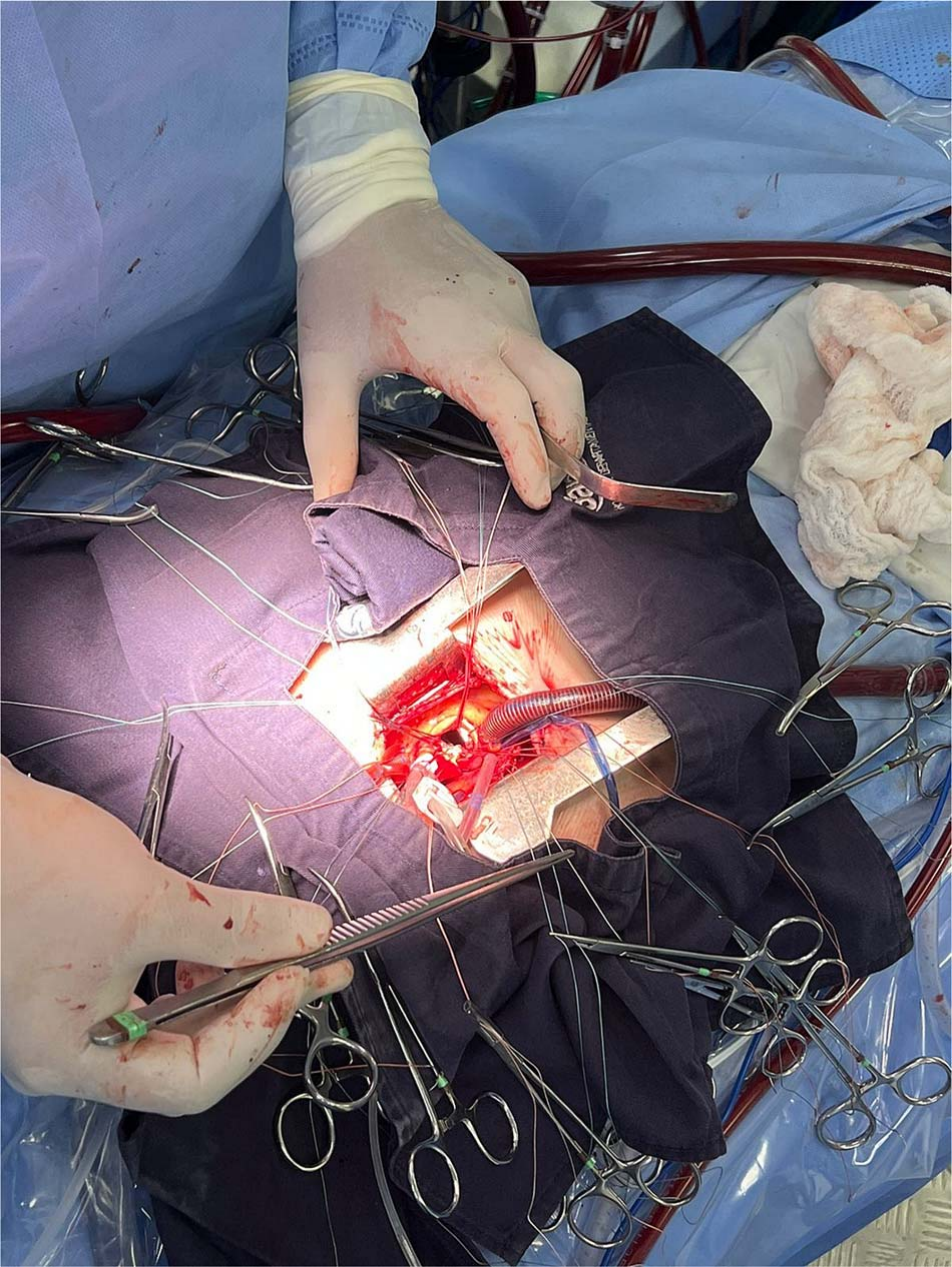
Mini-sternotomy.

**Figure 2:**
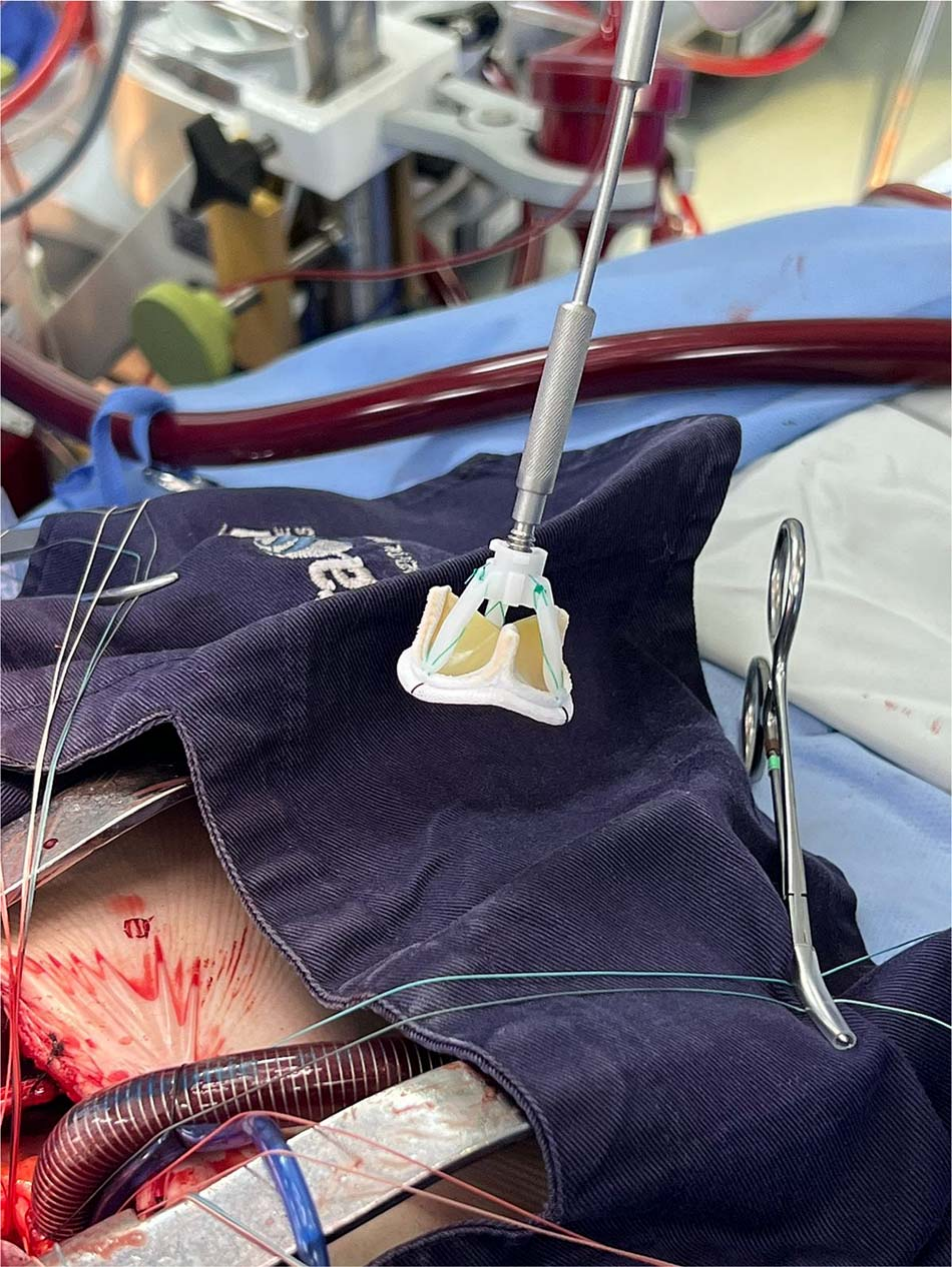
Bioprostheses Inspiris Resilia®.

The patient underwent minimally invasive cardiac surgery (Fig. 1) through an upper mini-sternotomy, with valve replacement using a 25-mm Inspiris Resilia® bioprosthesis (Fig. 2) and aortic reconstruction with a pericardial patch. Extracorporeal circulation lasted 107 minutes with an aortic cross-clamp time of 100 minutes. No intraoperative complications occurred, and he was transferred to the intensive care unit.

**Figure 3:**
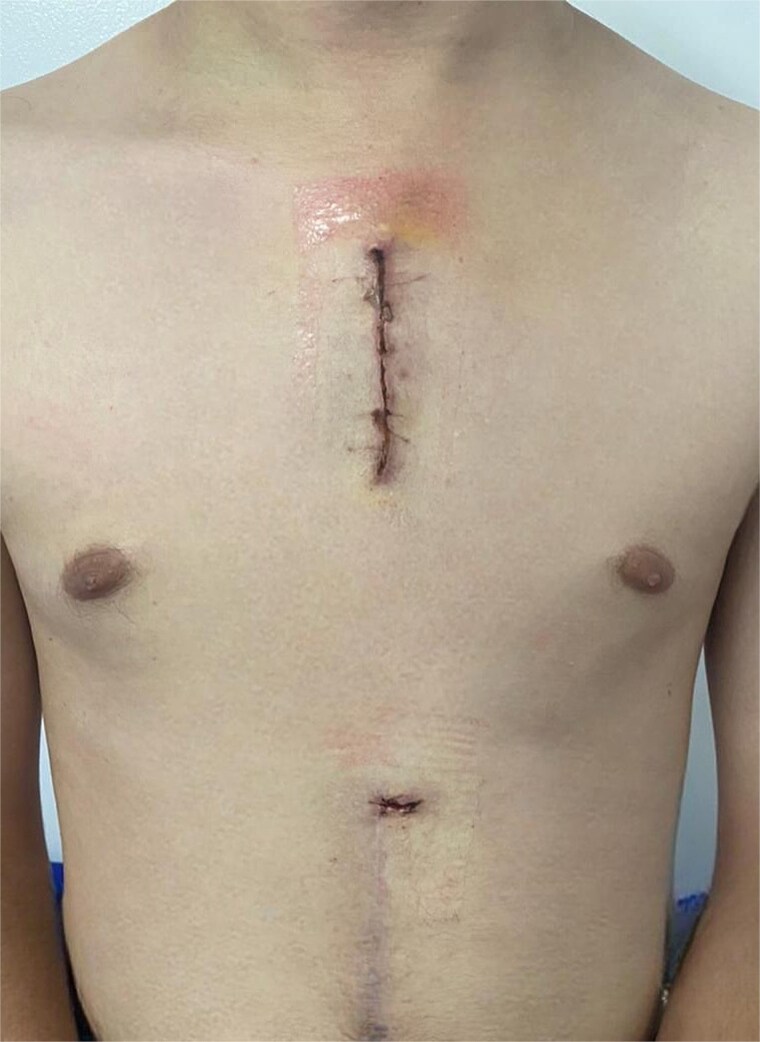
Outpatient follow-up.

During his ICU stay, the patient had an adequate postoperative course. He did not require oxygen therapy after extubation, but did require vasodilator infusion for hypertension, later transitioned to oral antihypertensive therapy with good control. He was transferred to the general ward on postoperative day four, where the cardiology service adjusted medications, and given his stable condition, he was discharged with outpatient follow-up (Fig. 3).

## Discussion

We present the case of a young patient with Wunderlich syndrome and congenital BAV, complicated by severe AR that required early surgical intervention due to the high risk of cardiovascular complications.

In our patient, the BAV was classified as type 1 with right–left cusp fusion, the most common subtype, which has been strongly associated with AR [11]. The mechanism of regurgitation was cusp prolapse, a recognized cause of severe AR in this subtype, favored by asymmetric leaflet stress and progressive root dilation [12].

Wunderlich syndrome is defined as an acute, spontaneous, non-traumatic renal hemorrhage of idiopathic etiology [13]. In this case, valve prosthesis selection was carefully discussed with the patient. The use of a mechanical prosthesis was avoided due to the increased risk of recurrent major bleeding under anticoagulation. This decision follows ACC/AHA recommendations to consider bioprosthetic valves at any age in patients with contraindications to anticoagulation [8]. Newer surgical options, such as valve-in-valve procedures, offer a realistic alternative in the event of structural valve deterioration in the coming years.

The prosthesis used, the Inspiris Resilia® biological valve, is designed to reduce phospholipid content and detoxify glutaraldehyde, protecting tissue from calcification and degeneration [14]. This model also incorporates fluoroscopically visible markers and an expansion zone (Vfit) to facilitate future valve-in-valve implantation [14]. The COMMENCE trial demonstrated decreased thromboembolic events, bleeding, paravalvular leak, and pacemaker requirement, while improving valve area and mean gradient [15].

Although Inspiris Resilia® shows encouraging mid-term durability in older patients, such as those included in the COMMENCE trial (mean age 66 years) [15], its long-term performance in very young patients remains uncertain. Biological prostheses are known to degenerate faster in younger populations due to increased calcium metabolism and hemodynamic stress [16]. Alternatives such as valve repair or the Ross procedure should also be considered. Valve repair, when feasible, preserves native tissue and avoids prosthetic complications, though durability depends on morphology and surgical expertise [17]. The Ross procedure provides excellent hemodynamics and survival comparable to the general population, but requires highly specialized centers and carries a risk of autograft or right ventricular outflow tract reintervention [17].

Thus, lifetime management of aortic valve disease in young patients must balance the risks and benefits of repair, replacement with mechanical or biological prostheses, and the role of valve-in-valve reinterventions [18].

## Conclusion

Surgical management of valvular disease requires consideration of multiple options and prosthesis types, weighing advantages, disadvantages, and strategies to minimize complications. In young patients, the goal is to preserve quality of life while anticipating the need for future reinterventions.

Minimally invasive cardiac surgery should be more widely implemented given its advantages. In this case, it reduced hospital stay, morbidity, and complications typically associated with conventional approaches.

## Data Availability

No datasets were generated or analyzed during the current study.
